# Supplementary material for: MetaFlowTrain: a highly parallelized and modular fluidic system for studying exometabolite-mediated inter-organismal interactions
Source: Nat Commun. 2025 Apr 10;16:3310. doi: 10.1038/s41467-025-58530-x (PMC11985495; doi:10.1038/s41467-025-58530-x)
Supplement: Supplementary file 1 — Supplementary Information [file 41467_2025_58530_MOESM1_ESM.pdf]

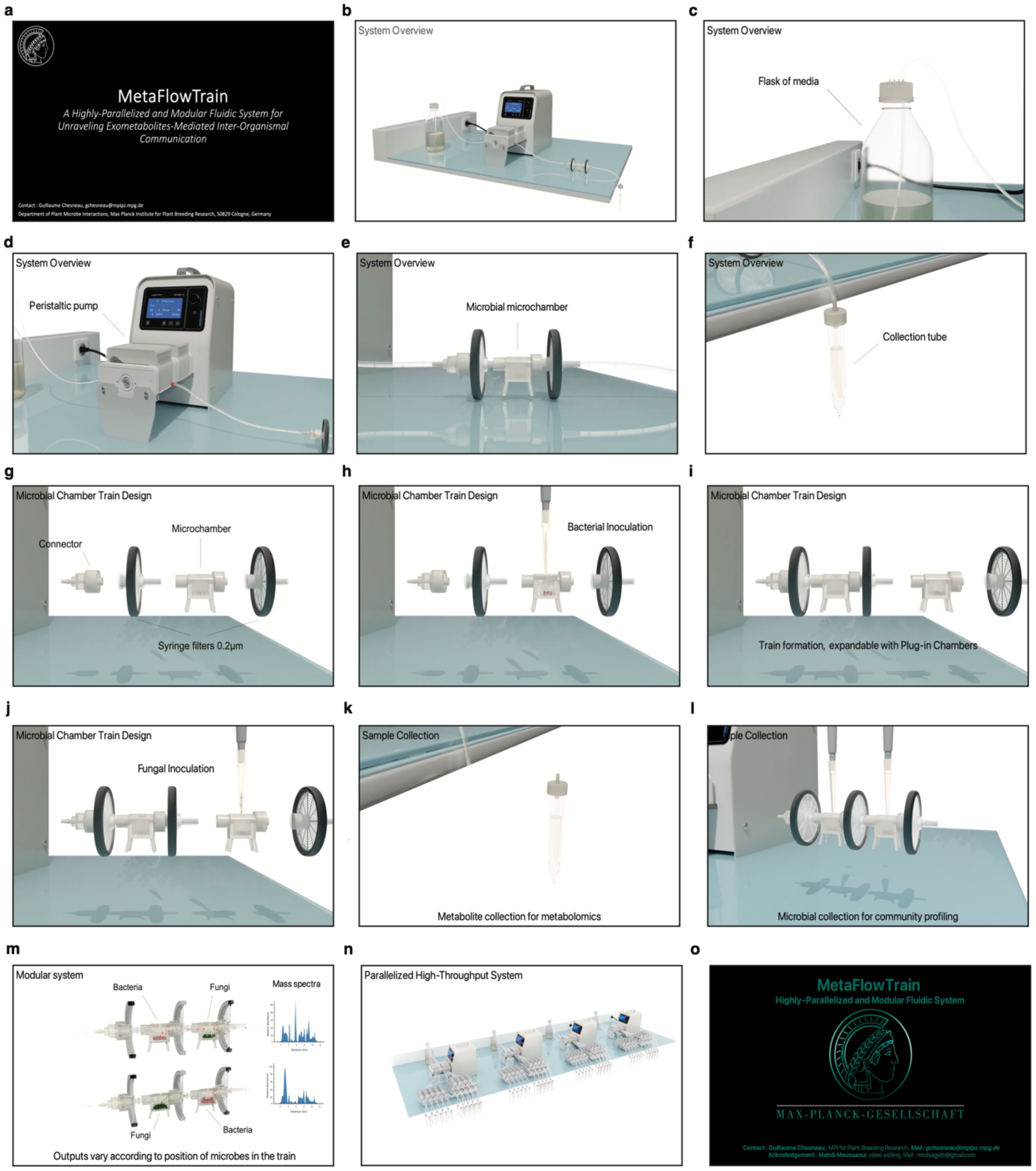

Supplementary Fig. 1: Visual summary of "Supplementary Movie 1". This figure presents key sections from the movie, arranged chronologically. **a–f)** Overview of the system; **g)** Structure of a microbial microchamber train; **h–j)** Inoculation of the microchambers and the modular train system; **k–l)** Collection of exometabolites and microbes; **m)** Schematic of exometabolite exchange between trains of two microchambers; **n–o)** Overview of the system with four pumps running in parallel, resulting in 96 samples.

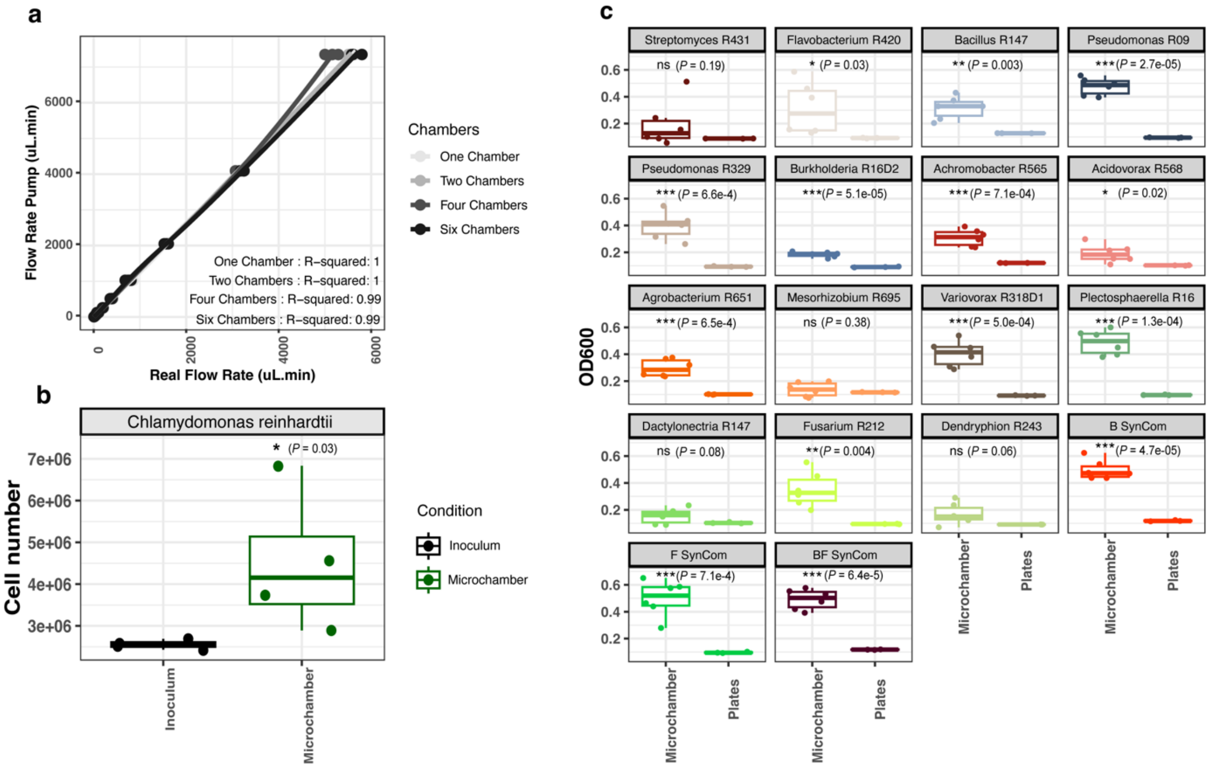

Supplementary Fig. 2: MetaFlowTrain growth capacities. a) Linear Regression analysis (OLS estimation) of the flow rate set on the pump (y-axis) vs. the actual flow rate observed (x-axis). Colors represent different microchamber configurations (1, 2, 4, or 6 microchambers per train). n = 3 independent microchamber trains per treatment. Model fit was assessed using R^2 for each configuration. **b)** Boxplots showing the number of *Chlamydomonas reinhardtii* cells in the inoculum and after 62 hours of growth (x-axis). Boxplots are delimited by the first and third quartiles, with the central line representing the median value. Whiskers extend to show the range of the data within 1.5 * IQR from the quartiles, and all data points are displayed as individual points, including outliers. n = 4 independent inoculum and n = 4 independent microchamber per treatment. Significance, compared to the Inoculum, was determined with two-sided Wilcoxon test (* $P < 0.05$). **c)** Growth of 11 bacterial strains, four fungi, and their respective SynComs. Cultures were grown for 62 hours in peat extract in the MetaFlowTrain or 96-well plates (x-axis). Microbial growth (OD_{600}) is shown on the y-axis. n = 3 independent inoculum per treatment for “Plates” and n = 6 independent microchamber per treatment for “Microchamber”. Significant differences compared to inoculum OD was determined with two-sided two-sided t-tests (* $P < 0.05$, ** $P < 0.01$, *** $P < 0.001$). Colors represent the different strains. Source data are provided as a Source Data file.

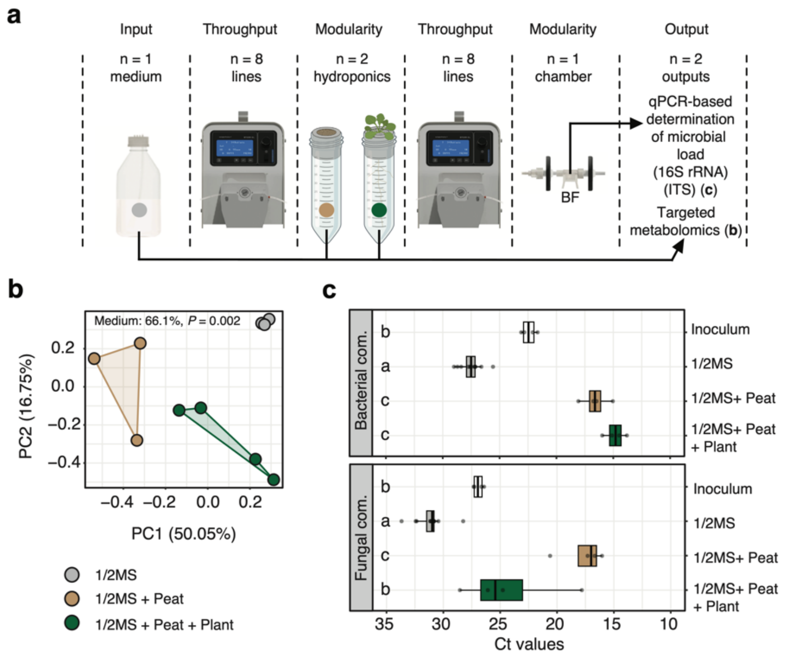

Supplementary Fig. 3: Influence of *Arabidopsis* root exudates on microbial growth. a) Schematic of the MetaFlowTrain setup, which includes ½ MS medium fed into two hydroponic systems (planted in green, non-planted in beige). Each system connects to a microchamber. Outputs: microbial load (qPCR) and exometabolic profiles (targeted). BF: Bacterial–Fungal SynCom. Hydroponic system was created in BioRender (Herpell, J. (2025) https://BioRender.com/r27z552). **b)** PCA displaying exometabolite profiles from microchambers running with medium through peat (beige, n = 3 independent microchamber + hydroponic system) or planted peat substrate (green, n = 4 independent microchamber + hydroponic system). Statistical significance was determined by PERMANOVA-based comparison of metabolic profiles (EuclideanDistances~Medium, permutations = 999). **c)** Boxplots showing microbial load (*Ct* values) for SynComs (panels) inoculated in microchambers. Boxplots are delimited by the first and third quartiles, with the central line representing the median value. Whiskers extend to show the range of the data within 1.5 * IQR from the quartiles, and all data points are displayed as individual points, including outliers. n = 4 independent microchamber + hydroponic system per treatment. Letters denote statistically significant differences in microbial abundances (two-sided ANOVA, and *post-hoc* Tukey's test, $P < 0.05$). Source data are provided as a Source Data file.

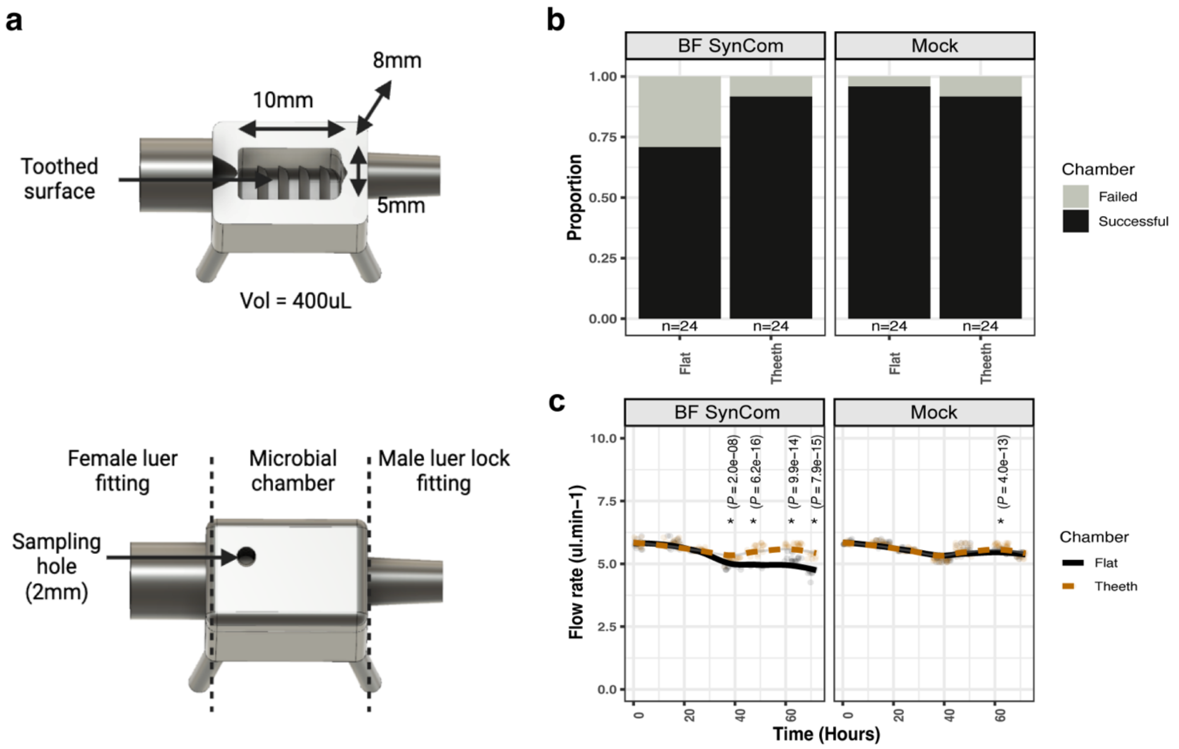

Supplementary Fig. 4: Microchamber design. a) Top view of a microchamber with (top) or without (bottom) a horizontal cross-section. **b)** Bar chart indicating the success rate of microchambers with flat or toothed bottoms (x-axis), when inoculated with microbes (BF SynCom) or without (MF). n=24 individual microchamber per condition **c)** Flow rate over time in microchambers inoculated with the Bacterial–Fungal SynCom (BF) or left non-inoculated (Mock). Colors represent the two microchambers designs. n=24 individual microchamber per condition. Significance, between microchambers for each time point, was determined with two-sided t-tests (* $P < 0.05$). Source data are provided as a Source Data file.

Supplementary Table 1: Microbial strains used in the study

IDs		Taxonomy						
ID	Full_ID	Kingdom	Phylum	Class	Order	Family	Genus	Origin
R09	Pseudomonas R09	Bacteria	Proteobacteria	Gammaproteobacteria	Pseudomonadales	Pseudomonadaceae	Pseudomonas	Bai *et al*. 2015
R16D2	Burkholderia R16D2	Bacteria	Proteobacteria	Betaproteobacteria	Burkholderiales	Comamonadaceae	Methylibium	Bai *et al*. 2016
R147	Bacillus R147	Bacteria	Firmicutes	Bacilli	Bacillales	Bacillaceae	Bacillus	Bai *et al*. 2017
R318D1	Variovorax R318D1	Bacteria	Proteobacteria	Betaproteobacteria	Burkholderiales	Comamonadaceae	NA	Bai *et al*. 2018
R329	Pseudomonas R329	Bacteria	Proteobacteria	Gammaproteobacteria	Pseudomonadales	Pseudomonadaceae	Pseudomonas	Bai *et al*. 2019
R420	Flavobacterium R420	Bacteria	Bacteroidetes	Flavobacteriia	Flavobacteriales	Flavobacteriaceae	Flavobacterium	Bai *et al*. 2020
R431	Streptomyces R431	Bacteria	Actinobacteria	Actinobacteria	Actinomycetales	Streptomycetaceae	Streptomyces	Bai *et al*. 2021
R565	Achromobacter R565	Bacteria	Proteobacteria	Betaproteobacteria	Burkholderiales	Alcaligenaceae	NA	Bai *et al*. 2022
R568	Acidovorax R568	Bacteria	Proteobacteria	Betaproteobacteria	Burkholderiales	Comamonadaceae	NA	Bai *et al*. 2023
R651	Agrobacterium R651	Bacteria	Proteobacteria	Alphaproteobacteria	Rhizobiales	Rhizobiaceae	Agrobacterium	Bai *et al*. 2024
R695	Mesorhizobium R695	Bacteria	Proteobacteria	Alphaproteobacteria	Rhizobiales	Phyllobacteriaceae	NA	Bai *et al*. 2025
R401	Pseudomonas R401	Bacteria	Proteobacteria	Gammaproteobacteria	Pseudomonadales	Pseudomonadaceae	Pseudomonas	Bai *et al*. 2025
R401	R401 ΔphldΔpvdyΔsypc	Bacteria	Proteobacteria	Gammaproteobacteria	Pseudomonadales	Pseudomonadaceae	Pseudomonas	Getzke et al, 2024
F016	Plectosphaerella R16	Fungi	Ascomycota	Sordariomycetes	Hypocreomycetidae	Plectosphaerellaceae	Plectosphaerella	Mesny et al. 2021
F147	Fusarium R212	Fungi	Ascomycota	Sordariomycetes	Hypocreales	Nectriaceae	Fusarium	Mesny et al. 2021
F212	Dendryphion R243	Fungi	Ascomycota	Dothideomycetes	Pleosporales	Pleosporaceae	Dendryphion	Mesny et al. 2021
F243	Dactylonectria R147	Fungi	Ascomycota	Ascomycota	Ascomycota	Ascomycota	Dactylonectria	Mesny et al. 2021
Cr	Chlamydomonas reinhardtii CC1690	Plantae	Chlorophyta	Chlorophyceae	Chlamydomonadales	Chlamydomonadaceae	Chlamydomonas	Durán et al., 2022

Supplementary Table 2: Estimation of the costs of one MetaFlowTrain experiment

Information from the company							
**Parts**	**Product**	**Reference**	**Company**	**Number per Box**	**Unit**	**Price from company**	**Date price**
Microchambers	Biomed Amber Resin	RS-F2-BMAM-01	3Dmensionals	1	Liter	294.91	06.09.24
Connector	Biomed Amber Resin	RS-F2-BMAM-01	3Dmensionals	1	Liter	294.91	06.09.24
Falcon_Cap	Biomed Amber Resin	RS-F2-BMAM-01	3Dmensionals	1	Liter	294.91	06.09.24
Duran_Bottle_Cap	Biomed Amber Resin	RS-F2-BMAM-01	3Dmensionals	1	Liter	294.91	06.09.24
Rack 1 microchamber	Clear Resin V4	RS-C2-GPCL-05	3Dmensionals	1	Liter	177.31	06.09.24
Rack 2 microchamber	Clear Resin V4	RS-C2-GPCL-05	3Dmensionals	1	Liter	177.31	06.09.24
Filters	0,22 µm Spritzenvorsatzfilter, PVDF (steril), blau, Durchmesser: ⌀33 mm	E4780-1221	StarLab	100	Units	40.125	06.09.24
Silicon rings	RS PRO O-Ring Silikon, Innen-Ø 3.68mm / Außen-Ø 9/32Zoll, Stärke 1.78mm, 1 Beutel mit 50 Stück	527-9740	RS	50	Units	7.66	06.09.24
**Information for the MetaFlowTrain**							
**Form print file**	**Quantity: One Train of 1 microchamber**	**Quantity: One Train of 2 microchambers**	**Volume Resin**	**Price per unit (eu**	**Timelif**	**Price 1 sample (1 microchamber)**	**Price 1 sample (2 microchambers)**
MetaFlowTrain_microchambers_60.form	1	2	3.0965	0.913188815	2-3 use	0.913188815	1.82637763
MetaFlowTrain_Connectors.form	1	2	0.993454545	0.29297968	2-3 use	0.29297968	0.58595936
15mL_Falcon_Cap.form	1	1	3.680666667	1.085465407	Unlimi	1.085465407	1.085465407
Duran_Bottle_Cap.form	0.041666667	0.041666667	28.8225	8.500043475	Unlimi	0.354168478	0.354168478
Rack_1_Microchamber.form	0.2	0.2	55.0275	9.756926025	Unlimi	1.951385205	1.951385205
Rack_2_Microchambers.form	0.2	0.2	72.7	12.890437	Unlimi	2.5780874	2.5780874
NA	2	3	NA	0.40125	Single	0.8025	1.20375
NA	2	3	NA	0.1532	Single	0.3064	0.4596

Supplementary Table 3: List of compounds used for targeted metabolomics analysis

KEGG	Chebi ID	Standard Name (Chebi)	Vendor Name	Compound family	Brand	Order number
NA	NA	NA	Amino acids Mix standard Metabolomics _Labelled	Mix	CIL	MSK-A2-1.2
C00062	CHEBI:16467	L-arginine	Arginine	Amino Acids	Sigma	A5131
C00041	CHEBI:16977	L-alanine	L-Alanine	Amino Acids	Sigma	A7627
C00152	CHEBI:17196	L-asparagine	L-Asparagine	Amino Acids	Sigma	A0884
C00025	CHEBI:16015	L-glutamic acid	L-Glutamic acid	Amino Acids	Sigma	G1251
C00123	CHEBI:15603	L-leucine	L-Leucine	Amino Acids	Sigma	L8000
C00047	CHEBI:18019	L-lysine	L-Lysine	Amino Acids	Sigma	L5626
C00077	CHEBI:15729	L-ornithine	L-Ornithine monohydrochloride	Amino Acids	Sigma	O2375
C00079	CHEBI:17295	L-phenylalanine	L-Phenylalanine	Amino Acids	Sigma	P2126
C00148	CHEBI:17203	L-proline	L-Proline	Amino Acids	Sigma	P0380
C00065	CHEBI:17115	L-serine	L-Serine	Amino Acids	Sigma	S4500
C00188	CHEBI:16857	L-threonine	L-Threonine	Amino Acids	Sigma	T8625
C00078	CHEBI:16828	L-Tryptophane	L-Tryptophane	Amino Acids	Sigma	T0254
C00082	CHEBI:17895	L-tyrosine	L-Tyrosine	Amino Acids	Sigma	T3754
C00183	CHEBI:16414	L-valine	Valine	Amino Acids	Sigma	V0500
C00064	CHEBI:18050	L-glutamine	Glutamine	Amino Acids	Sigma	G3126
C00037	CHEBI:15428	glycine	Glycine	Amino Acids	Sigma	G7126
C00135	CHEBI:15971	L-histidine	Histidine	Amino Acids	Sigma	H8126
C00049	CHEBI:17053	L-aspartic acid	Aspartic acid	Amino Acids	Sigma	A9256
C00334	CHEBI:16865	gamma-aminobutyric acid	γ- Aminobutyric Acid (GABA)	Amino Acids	Sigma	A2129
C00417	CHEBI:32805	cis-aconitic acid	Cis - Aconitic acid	TCA-Glycolysis	Sigma	A3412
C00158	CHEBI:30769	citric acid	Citric acid anhydrous	TCA-Glycolysis	Sigma	251575
C00392	CHEBI:16899	D-mannitol	D-(-)-Mannitol	TCA-Glycolysis	Sigma	M4125
C00221	CHEBI:15903	beta-D-glucose	D-(+)-Glucose	TCA-Glycolysis	Supelco	47249
C00095	CHEBI:15824	D-fructose	D-fructose	TCA-Glycolysis	Sigma	F2793
C00257	CHEBI:33198	D-gluconic acid	D-gluconic acid	TCA-Glycolysis	Sigma	64188
C01432	CHEBI:28358	rac-lactic acid	DL-Lactic acid	TCA-Glycolysis	Supelco	PHR1215
C00122	CHEBI:18012	fumaric acid	Fumaric acid	TCA-Glycolysis	Sigma	47910
C00149	CHEBI:30797	(S)-malic acid	Malic acid	TCA-Glycolysis	Sigma	94916
C00093	CHEBI:15978	sn-glycerol 3-phosphate	sn - Glycerol - 3- phosphate	TCA-Glycolysis	Sigma	94124
C00042	CHEBI:15741	succinic acid	Succinic acid	TCA-Glycolysis	Sigma	S3674
C00089	CHEBI:17992	Sucrose	Sucrose	TCA-Glycolysis	Sigma	S7903
C00026	CHEBI:30915	2-oxoglutaric acid	α-Ketoglutaric acid disodium salt hydrate	TCA-Glycolysis	Sigma	K3752
C00661	CHEBI:17138	glyceraldehyde 3-phosphate	DL-Glyceraldehyde 3-phosphate solution 45-55 mg/mL in H2O	TCA-Glycolysis	Sigma	G5251
C00022	CHEBI:32816	pyruvic acid	Sodium Pyruvate	TCA-Glycolysis	Sigma	P2256
C00258	CHEBI:32398	D-glyceric acid	Glyceric acid	TCA-Glycolysis	Sigma	61786

Supplementary Protocol 1: PDF protocol exported from protocol.io (V3, DOI : dx.doi.org/10.17504/protocols.io.36wgqd68ovk5/v3)

Mar 14, 2025 Version 3

Assembly and sample collection in the MetaFlowTrain V.3

Version 1 is forked from Assembly and sample collection in the MetaFlowTrain

DOI
dx.doi.org/10.17504/protocols.io.36wgqd68ovk5/v3

Guillaume Chesneau[1]

[1]Department of Plant Microbe Interactions, Max Planck Institute for Plant Breeding Research, 50829 Cologne, Germany.

Guillaume Chesneau

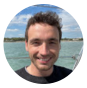

Guillaume Chesneau
Max Planck Institute for Plant Breeding Research

OPEN ACCESS

DOI: dx.doi.org/10.17504/protocols.io.36wgqd68ovk5/v3

Protocol Citation: Guillaume Chesneau 2025. Assembly and sample collection in the MetaFlowTrain. **protocols.io** https://dx.doi.org/10.17504/protocols.io.36wgqd68ovk5/v3Version created by **Guillaume Chesneau**

License: This is an open access protocol distributed under the terms of the **Creative Commons Attribution License**, which permits unrestricted use, distribution, and reproduction in any medium, provided the original author and source are credited

Protocol status: Working
We use this protocol and it's working

Created: December 19, 2024

Last Modified: March 14, 2025

Protocol Integer ID: 124409

Keywords: Metabolomics, Cell communication, microfluidics, bioreactor, microbe-microbe interactions, microbe-host interaction

Abstract

The following protocol is for the preparation of a MetaFlowTrain experiment. It provides the required materials, and the procedure. We also have a video protocol to assist you in handling the MetaFlowTrain
We welcome all input to improve the system and are eager to craft new adapters/microchambers tailored to your scientific needs.

https://www.youtube.com/embed/BYc7E3fybTI?si=bp1FkvV4Uy8DYm54

Materials

METAFLOWTRAIN 3D PRINTED MATERIALS

Certain materials need to be 3D printed before starting the experiments. The following objects can be printed in advance and stored in a dark, dry area. The quantity of 3D-printed items can be adjusted according to the specific number of pieces required.

All the files below in the '.form' format can be directly uploaded to a 3D printer interface (e.g., Preform, Formlabs).

- **Biomed Ambers Resin (Formlabs)**

- Microchambers (Model: *MetaFlowTrain_microchambers_60.form*) FILE MetaFlowTrain_microchambers_60.f… 7.8MB
- Connectors (Model: *MetaFlowTrain_Connectors.form*)

FILE MetaFlowTrain_Connectors.form 281KB

- Duran bottle adapters (Model: *Duran_Bottle_Cap.form*)

FILE Duran_Bottle_Cap.form 4.1MB

- Falcon 15ml adapters (Model: *15mL_Falcon_Cap.form*)

FILE 15mL_Falcon_Cap.form 716KB

- **Clear Resin (Formlabs)**

- Rack for 1 microchamber train (Model: Rack_1_Microchamber.form)

FILE Rack_1_Microchamber.form 3.1MB

- Racks for 2 microchambers train (Model: Rack_2_Microchamber.form)

FILE Rack_2_Microchambers.form 1.4MB

In supplementary, we provide all the .stl file.

- Microchambers (Model: *MetaFlowTrain_microchamber.stl*)

FILE MetaFlowTrain_microchamber.stl 906KB

- Connectors (Model: *MetaFlowTrain_Connector.stl*)

FILE MetaFlowTrain_Connector.stl 539KB

- Duran bottle adapters (Model: Duran_Bottle_Cap.*stl*)

FILE Duran_Botlle_Cap.stl 1.4MB

- Falcon 15ml adapters (Model: *15mL_Falcon_Cap.stl*)

FILE 15mL_Falcon_Cap.stl 1.8MB

- Rack for 1 microchamber train (Model: Rack_1_Microchamber.stl)

FILE Rack_1_Microchamber.stl 583KB

- Racks for 2 microchambers train (Model: Rack_2_Microchamber.stl)

FILE Rack_2_Microchambers.stl 926KB

OTHER METAFLOWTRAIN MATERIALS

- **Peristaltic pump**

- (LG-BT100-1L-A-EU/DG-24-A, Darwin Microfluidics)

- **Syringe filters**

- 0,22 µm Spritzenvorsatzfilter, PVDF (steril), blau, Durchmesser: ⌀33 mm (Starlab, E4780-1221)

- **Biocompatible Tape**

- Biocompatible tape (Polyolefin Diagnostic Tape, Nr 9793R, 3M)

- **Hoses**

- 3-stop Platinum-cured Silicone Tubing: SE-TUB-SIL-SSS-1*1 (Darwin microfluidics)
- Platinum-cured Silicone Tubing (15m): SE-TUB-SIL-1*1 (Darwin microfluidics)
- Tygon E-LFL Pump Tubing (7.5m): SA-AVX42007, (Darwin Microfluidics)

- **Silicon rings**

- RS PRO O-Ring Silikon, Innen-Ø 3.68mm / Außen-Ø 9/32Zoll, Stärke 1.78mm, 1 Beutel mit 50 Stück
- RS PRO O-Ring FKM, Innen-Ø 2mm / Außen-Ø 4mm, Stärke 1mm

Procedure

1 **DAY 1 : PREPARE MATERIAL AND BUILD MICROCHAMBER TRAINS**

1.1 **1.1 PREPARE MATERIAL :**

1h

- **1.1.1 Cut 1cm-wide strips of biocompatible tape (Polyolefin Diagnostic Tape, Nr 9793R, 3M) with precuts every 2cm (Figure 1) and store it in a container (e.g. glass beaker).**

Comment: This tape cannot be autoclaved, which is not a problem since *the sticky side is sterile.* however*, we will still UV sterilize the surfaces later to maintain a sterile environment while handling the MetaFlowTrain system.*

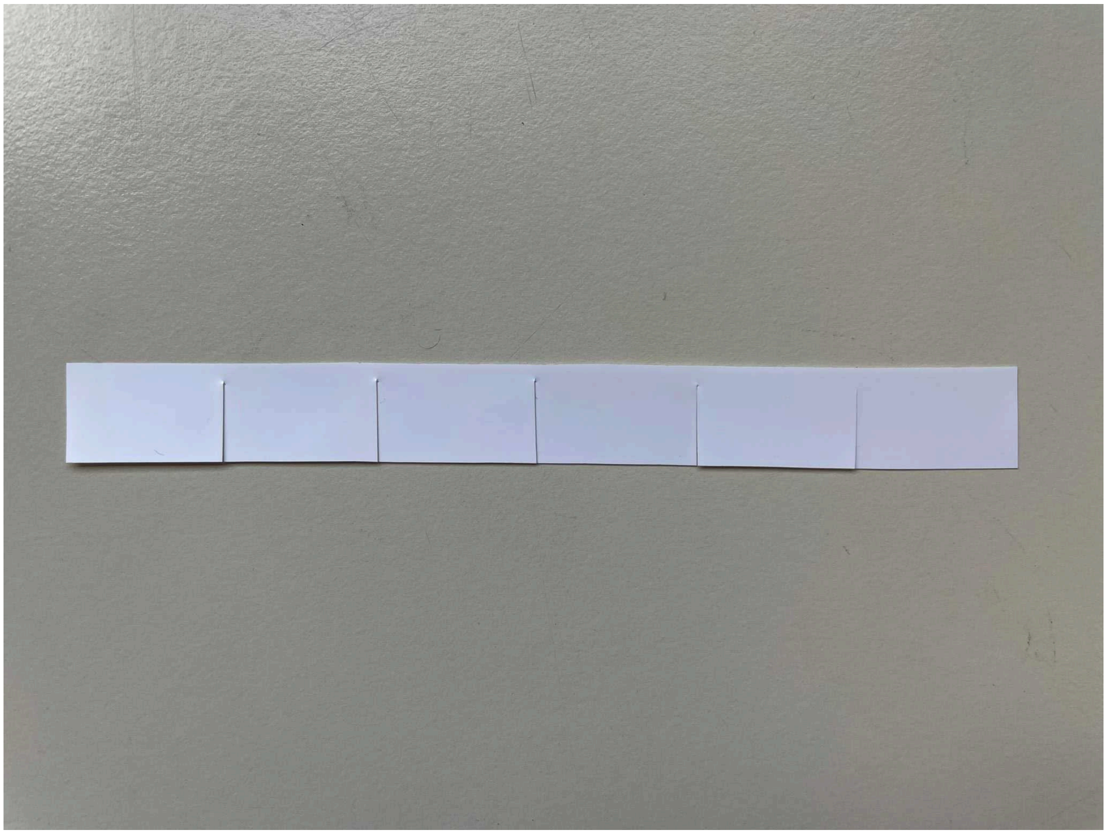

- **Figure 1:** Pre-cut biocompatible tape (Polyolefin Diagnostic Tape, No. 9793R, 3M)

- **1.1.2 Prepare and sterilize your media.**

Tip: ensure your media is already in the container you will use for your MetaFlowTrain experiment.

- **1.1.3 Sterilize the necessary number of microchambers and connectors.**

Comment: All 3D-printed pieces can be autoclaved for sterilization, as the resin is autoclavable.
Tip: Place the microchamber end connectors in separate boxes to help keep the bench organized during building. Boxes on the *video protocol: 0:28.*

- **1.1.4 Sterilize one box to store the opened sterile filters during the building of microchamber trains.**

Comment: Box on the video protocol: 0:25-0:31.

- **1.1.5 Sterilize boxes to store the final built microchamber trains**

Comment: Box on the video protocol: 0:28.

- **1.1.6 Assemble the 15 ml Falcon tube adapters by connecting the tubing (Tygon E-LFL Pump Tubing: SA-AVX42007 from Darwin Microfluidics). Place the assembled 15 ml Falcon tube adapters (Figure 2) in a box (*Box on the video protocol: 4:07)* and sterilize them by autoclaving.**

Comment : The length of the tubing can vary depending on your specific setup but should be consistent (e.g., 12 cm for 15mL Falcon tube adapters)

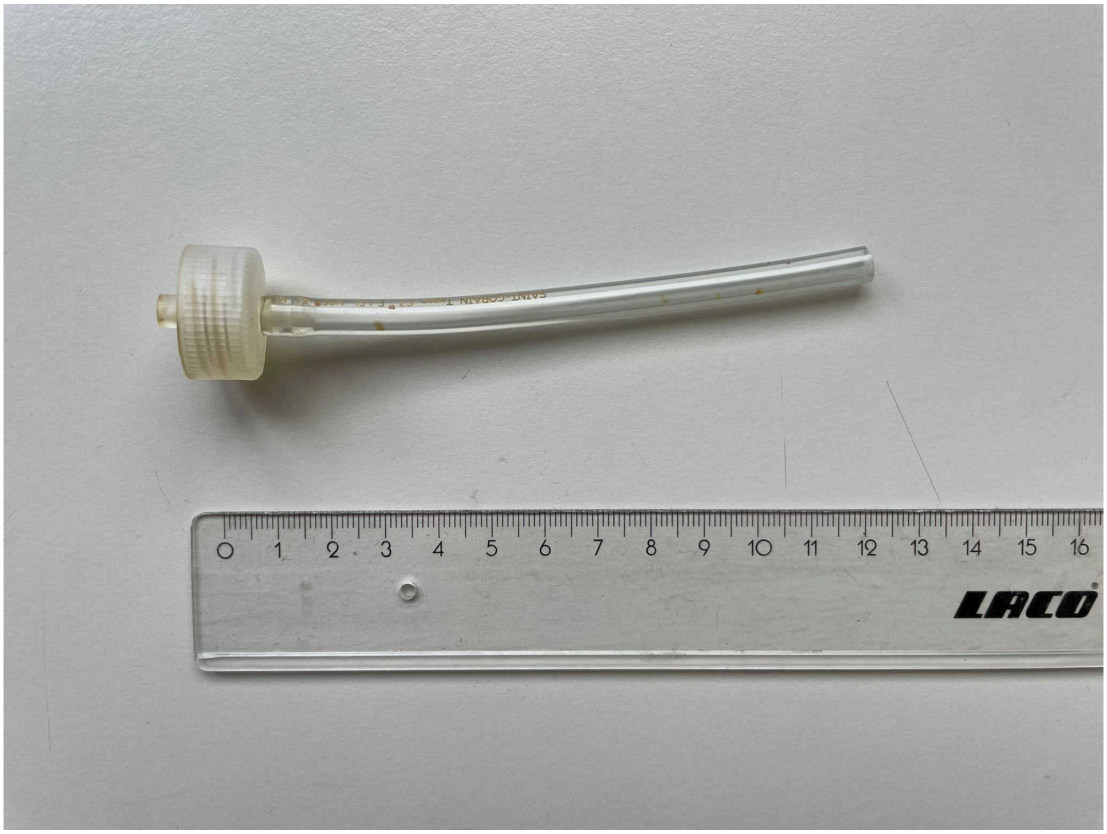

Figure 2: 15 mL Falcon tube adapter

- **1.1.7 Prepare the Duran bottle adapters by connecting the tubing (Platinum-cured Silicone Tubing: SE-TUB-SIL-1*1, Darwin Microfluidics). Place the assembled Duran bottle adapters (Figure 3) in a box (*Box on the video protocol: 2:24)* and sterilize (autoclave) them.**

Comment : The length of the tubing should be 22 cm for a 500 mL Duran bottle, but adjust it based on the type of bottle/Flacon you're using to ensure it touches the bottom of the bottle.

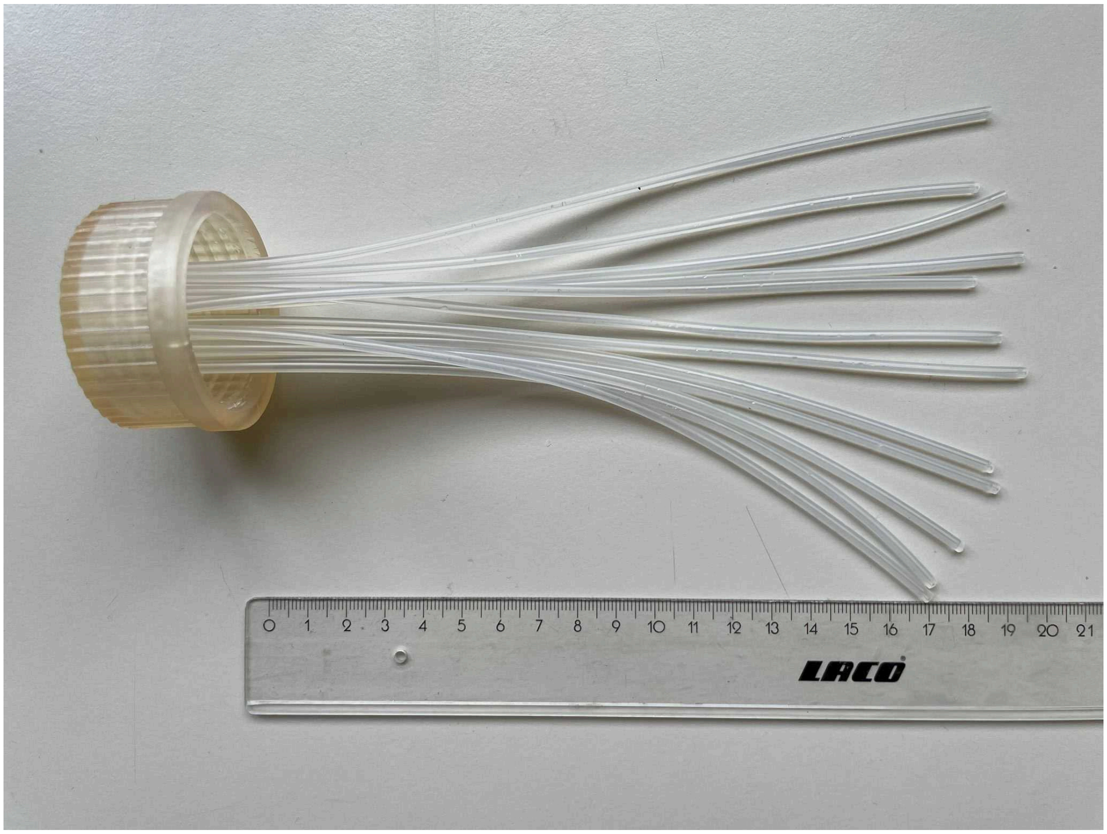

Figure 3 : Duran bottle adapter

- **1.1.8 Place silicon rings in a container and sterilize (autoclave).**

Comment: Beaker on the video protocol: 0:40.

- **1.1.9 (Additional) Place FKM rings in a container and sterilize (autoclave). FKM rings are necessary to ensure complete absence of leaks. See Troubleshooting 4.2 (Figure 8)**

1.2 **1.2. BUILDING MICROCHAMBER TRAINS (Video: 00:21:12) Video Protocol:** 1h

Comment before starting: *to ensure a complete absence of potential leaks, we recommend adding three autoclaved FKM rings to the male connector of the microchambers. This is not provided in the protocol video, they must be added in the male connector of the microchambers (video : 0:56 and 1:12) before plugging the filters in. Please see troubleshooting (4.2, figure 8) for details.*

- **1.2.1 Surface sterilize all equipment with Bacillol and expose it to UV light for 15-30 minutes. Items to be surface ster**

ilized include boxes with autoclaved microchambers and connectors, silicon rings, box for opened filters, and box for the final trains.

Comment: This process ensures the sterility of your working environment.

- **1.2.2 Unpack the filters and place them in a sterile box.**

Comment: (Video : 0:25-0:30).

- **1.2.3 Put a silicon ring in the connector and screw it to a syringe filter, ensuring a tight fit!!**

Comment: You should see pressure on the silicone ring (Video : 0:51-0:55).

- **1.2.4 Attach the microchamber, put a silicon ring in each female connector of the microchamber and connect it to another syringe filter (Figure 4)**

Comment: (Video : 0:57-1:04).

- **1.2.5 Place the completed trains in a sterile box**

Comment: (Video : 1:04).

- **1.2.6 You can repeat this step if you need a train of more than 1 microchamber.**

Comment: (Video : 1:05-1:26).

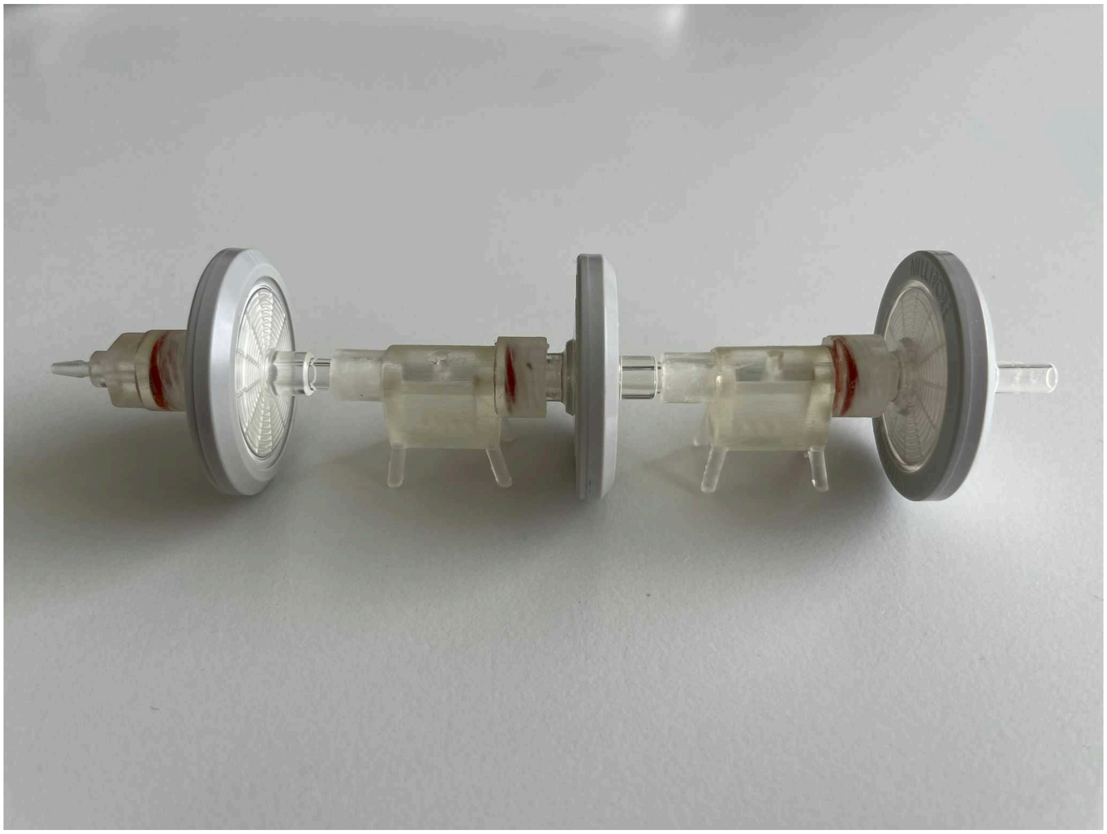

- **Figure 4 :** Completed train composed of two microchambers. ***See Figure 8, for the MetaFlowTrain version with FKM rings.***

Note

WARNING: Maintain a sterile environment throughout your experiment. Wash your gloves frequently to ensure sterility.

2 **DAY 2 : METAFLOWTRAIN EXPERIMENT**

2.1 **2.1 PREPARE YOUR MICROBIAL INOCULUM**

This step depends on your protocol.

We inoculate with 40uL of bacteria OD600, or 40uL of fungi, 2mg.mL.

Comment : The final volume in the microchamber is 400uL. Inoculating 40uL in the microchamber leads to a dilution to the tenth of your inoculum.

2.2 **2.2 PUMP TUBE CONNECTION AND STERILIZATION (Video: 01:38:00) Video Protocol:**

1h

- **2.2.1 Expose the pre-cut biocompatible tape (3M®, Figure 1) to UV light for at least 5 minutes on each side, then return it to sterile container.**
- **2.2.2 Pump tubes sterilization:**

a. Place the peristaltic pump on a clean bench.

b. Install the tubing (3-stop Platinum-cured Silicone Tubing: SE-TUB-SIL-SSS-1*1 (Darwin microfluidics) and tighten them to 7 notches.

Tip: can be done the day before (Video : 1:42-1:48).

c. Run 70% ethanol through the pump for 15minutes at speed 500uL.min. Then discard the Ethanol.

Comment : (Video : 1:48-1:59).

d. ***Run 500mL of water to clean the Ethanol, and empty the system.***

Comment : (Video : 2:02-2:17).

Tip : Always keep the tips of your tubing in a sterile container (e.g., a sterile beaker) to prevent contamination from touching your bench.

e. Carefully place the Duran bottle adapter (Figure 3) on your bottle filled with media and wrap it with Parafim or Micropore tape.

Comment : (Video : 2:20-2:42).

Note

WARNING : Don't tighten the Duran bottle adapter too much; you must keep the cap loose, and you might break the connectors.

f. Connect all the pump's tubing to the Duran bottle adapter.

Comment : (Video : 2:45-2:58).

Tip : If needed you can use a flat tweezers to help you adapting the tubing to the Duran cap adapter.

2.3 **2.3 MICROCHAMBER INOCULATION (Video: 03:00:00) Video Protocol:**

1h

- **2.3.1 Position your trains on racks (Figure 5)**

Comment : (Video : 3:03-3:10).

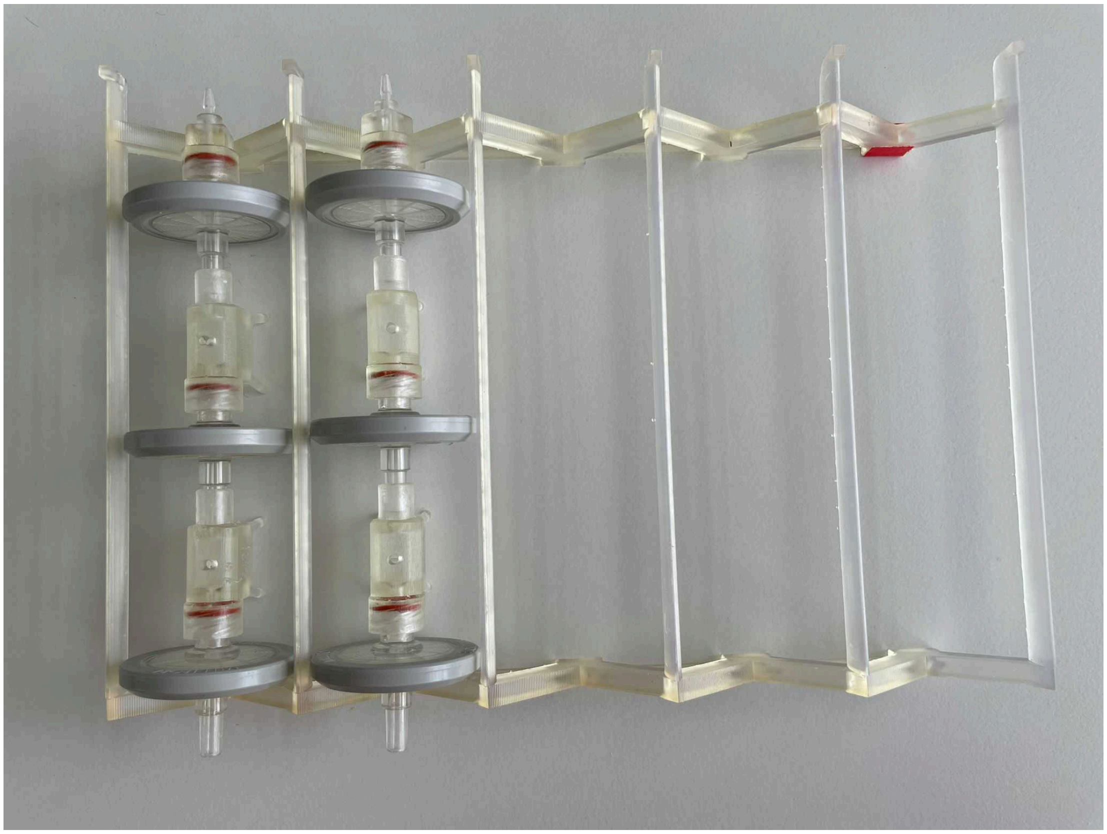

Figure 5: Example of two trains composed of two microchambers on a rack.

- **2.3.2 Add 40ul of the strain into your microchambers (your inoculum will be diluted 10 times in the microchamber).**

Comment : (Video : 3:11-3:23).

- **2.3.3 Seal the microchamber with biocompatible tape (3M®, Figure 1).**

Comment : (Video : 3:26-3:48).

Tip: Securely seal the tape with a PCR plate sealer to prevent leaks.

2.4 **2.4. CONNECT THE METAFLOWTRAIN (Video: 03:50:00) Video Protocol:** 1h

- **2.4.1 Connect the trains to the pump's tubes (3-stop Platinum-cured Silicone Tubing: SE-TUB-SIL-SSS-1*1 (Darwin microfluidics))**

Comment : (Video : 3:54-4:06).

- **2.4.2 Connect the tubes with the 15mL Falcon adapter to the end of the train**

Comment : (Video : 4:07-4:24).

- **2.4.3 Repeat these steps for the 24 channels of your pump.**
- **2.4.4 Screw 15mL Falcon tubes to the Falcon tube adapters, one per train (24 per pump).**

Comment : (Video : 4:07-4:24).

Note

WARNING: Don't tighten *them too much; you must keep the cap a bit loose.*

- **2.4.5 You're now ready to remove your pump from the clean bench and start your experiment !**

Comment: We are using a 90 cm x 60 cm plate with two handles to move the entire system from the clean bench to our growth chamber.

2.5 **2.5. START THE PUMP** 5m

- **2.5.1 Once all your pumps are prepared and settled in your growth chamber, fill the media at a high speed (e.g., 200 µL/min) until it reaches the first filters in the trains.**
- **2.5.2 Next, adjust the flow rate to a maximum of 20-50 µL/min to fill the microchambers.**

Comment: It's crucial to fill the microchambers slowly to avoid flushing your microbial inoculum into the filters.

- **2.5.3 When the liquid begins to exit the final filter in your train, set the pumps to your experimental flow rate (e.g., minimum flow rate = 7.349 µL/min).**

Tip: You can visually confirm that the microchambers are filling. Similarly, observe the filters; they will change color from dry (whiteish) to wet (grayish) as they become saturated. If a train fails to fill, abandon it and continue with your experiment.

- **2.5.4 Wait for the desired duration of the experiment.**

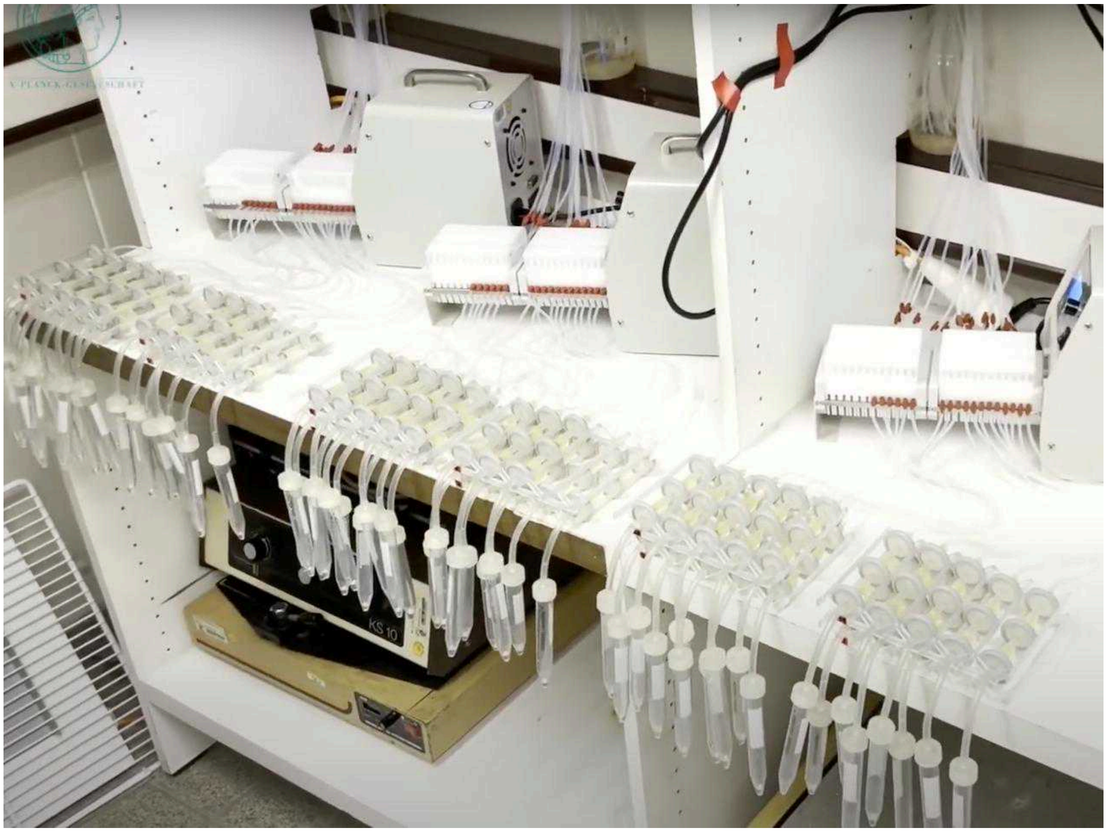

Figure 6: Example of a final MetaFlowTrain setup. This setup includes trains consisting of two microchambers, with four pumps running in parallel, resulting in 72 samples. Note that the MetaFlowTrain racks can be stacked to save space

Cleaning microchambers

3
- **3.1 After finishing your experiment, collect the microchambers and connectors in separate glass containers.**
- **3.2 Remove all the tape from the top of the microchambers.**
- **3.3 Fill the glass containers with water until all the parts are fully submerged.**
- **3.4 Place the glass containers in a sonicator for 15 minutes.**
- **3.5 Thoroughly wash the parts using a wash bottle with water, for microchambers, ensure the inside is thoroughly cleaned from the top hole.**
- **3.6 Let them dry completely.**
- **3.7 Store them dry in a box, in the dark, at room temperature until the next use.**

Note

WARNING: Carefully check for any cracks on your microchambers, as multiple autoclaving cycles can cause the resin to crack, especially if the microchambers are not properly dried before autoclaving.

TROUBLESHOOTING

4
- **4.1 Leaks on top of the microchambers:**

This is often caused by poor print quality of the microchambers, likely due to scratches or imperfections on their surface. Potential causes include:
1. Errors during the 3D printing process.
2. Insufficient drying of 3D prints before curing or autoclaving, which can cause cracks during heating and damage the prints.
3. Damage during removal of the prints from the support or platform.

Cracks on the microchambers can compromise their permeability. Refer to your printer's instructions and best practices to ensure high-quality prints.

- **4.2 Leaks between microchambers:**

On the screwed side: Leaks are typically caused by a loose connection. Ensure that the silicone rings are visibly compressed when screwing the microchambers or connectors with the syringe filters. While excessive force is not required, you should notice slight compression of the silicone ring to ensure a proper seal.

On the plugged side: Leaks may occur after multiple uses due to slight deformations of the microchambers. To prevent this issue, add 3 FKM rings (see Materials) inside the microchambers before inserting the filter. The filter will compress the FKM rings, ensuring a tight and secure seal. See the image below for reference (**Figure 8**).

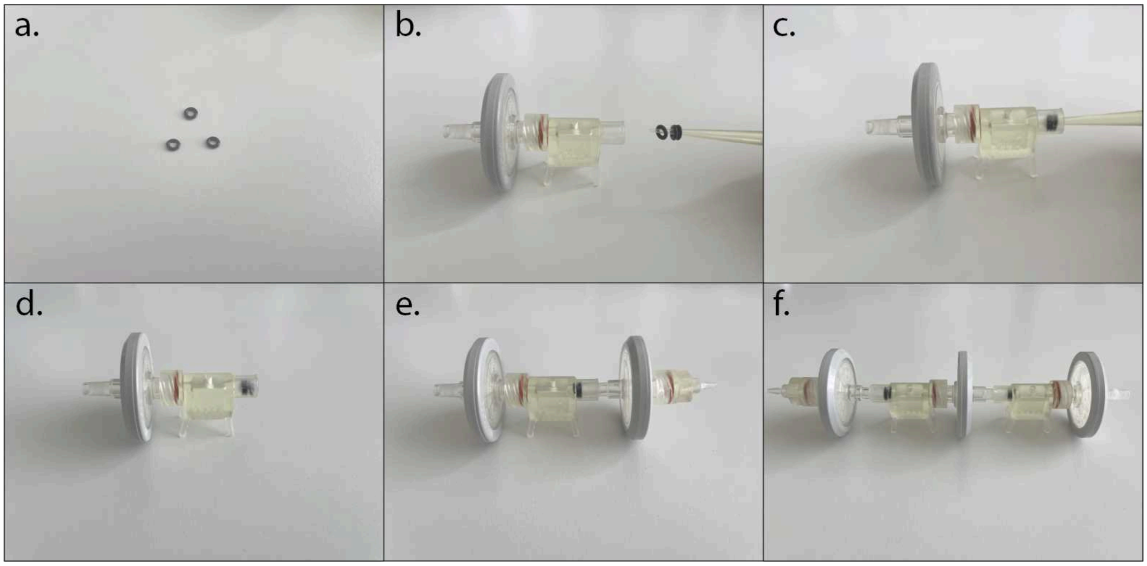

Figure 8: Adaptation of the MetaFlowTrain with FKM Rings. **a.** Three FKM rings (see Materials for details). **b.** Use a tip to pick up three FKM rings. **c-d.** Place the rings inside the microchamber. **e.** Insert the filter into the microchamber. **f.** Repeat as needed.

- **4.3 No media flowing into the hoses from the bottle:**

This may be caused by a loose connection to the bottle adapter. Check that no air is entering the connection, and ensure the hoses are fully plugged into the adapter. Also, inspect the adapter for any leftover 3D printing supports that could create air gaps and compromise the connection. In general, this issue often arises from poor print quality or improper cleaning/removal of supports from the print.

- **4.4 Air in the top of the microchambers:**

This is a common occurrence, as some air naturally passes through the MetaFlowTrain system. It's important to remove this air to ensure the microchambers are filled with liquid, especially before harvesting. To remove the air, tilt the microchambers to guide the air bubble toward the chamber's outlet. You can simply tilt the entire rack, and you'll notice the bubble moving toward the outlet. After a few minutes, the flow rate should push the air bubble out on its own.
