# Supplementary material for: MetaFlowTrain: a highly parallelized and modular fluidic system for studying exometabolite-mediated inter-organismal interactions
Source: Nat Commun. 2025 Apr 10;16:3310. doi: 10.1038/s41467-025-58530-x (PMC11985495; doi:10.1038/s41467-025-58530-x)
Supplement: Supplementary file 2 — Description of Additional Supplementary Information [file 41467_2025_58530_MOESM2_ESM.docx]

**Description of Additional Supplementary Files**

File Name: Supplementary Movie 1

Description: Movie of the MetaFlowTrain system.

File Name: Supplementary Movie 2

Description: Movie of the MetaFlowTrain system in operation in the lab.

File Name: Supplementary Movie 3

Description: Movie protocol for using the MetaFlowTrain system.

File Name: Supplementary Data 1

Description: 3D printing models (.form format).

File Name: Supplementary Data 2

Description: Stereolithography files (.stl format).

File Name: Supplementary Data 3

Description: Primers used in the study.

File Name: Supplementary Data 4

Description: Targeted metabolomics features for TCA cycle and glycolysis presented in Figure 2.

File Name: Supplementary Data 5

Description: Targeted metabolomics features for amino acids presented in figure 2.

File Name: Supplementary Data 6

Description: Targeted metabolomics features for TCA cycle and glycolysis presented in figure 3-4.

File Name: Supplementary Data 7

Description: Targeted metabolomics features for amino acids presented in figure 3-4.
