# Supplementary material for: MetaFlowTrain: a highly parallelized and modular fluidic system for studying exometabolite-mediated inter-organismal interactions
Source: Nat Commun. 2025 Apr 10;16:3310. doi: 10.1038/s41467-025-58530-x (PMC11985495; doi:10.1038/s41467-025-58530-x)
Supplement: Supplementary file 13 — Reporting Summary [file 41467_2025_58530_MOESM13_ESM.pdf]

nature portfolio

Corresponding author(s): HACQUARD Stéphane

Last updated by author(s): Mar 14, 2025

Reporting Summary

Nature Portfolio wishes to improve the reproducibility of the work that we publish. This form provides structure for consistency and transparency in reporting. For further information on Nature Portfolio policies, see our Editorial Policies and the Editorial Policy Checklist.

Statistics

For all statistical analyses, confirm that the following items are present in the figure legend, table legend, main text, or Methods section.

n/a	Confirmed	
☐	☒	The exact sample size (*n*) for each experimental group/condition, given as a discrete number and unit of measurement
☐	☒	A statement on whether measurements were taken from distinct samples or whether the same sample was measured repeatedly
☐	☒	The statistical test(s) used AND whether they are one- or two-sided<br>*Only common tests should be described solely by name; describe more complex techniques in the Methods section.*
☐	☒	A description of all covariates tested
☐	☒	A description of any assumptions or corrections, such as tests of normality and adjustment for multiple comparisons
☐	☒	A full description of the statistical parameters including central tendency (e.g. means) or other basic estimates (e.g. regression coefficient) AND variation (e.g. standard deviation) or associated estimates of uncertainty (e.g. confidence intervals)
☐	☒	For null hypothesis testing, the test statistic (e.g. *F*, *t*, *r*) with confidence intervals, effect sizes, degrees of freedom and *P* value noted<br>*Give P values as exact values whenever suitable.*
☒	☐	For Bayesian analysis, information on the choice of priors and Markov chain Monte Carlo settings
☐	☒	For hierarchical and complex designs, identification of the appropriate level for tests and full reporting of outcomes
☒	☐	Estimates of effect sizes (e.g. Cohen's *d*, Pearson's *r*), indicating how they were calculated

Our web collection on statistics for biologists contains articles on many of the points above.

Software and code

Policy information about availability of computer code

Data collection	No software was used to collect data
Data analysis	R version 4.2.2 was used to perform all analyses and vizualization. No statistical method was used to predetermine sample size. Amplicon sequencing data were analyzed using QIIME2 (v2023.2) and DADA2 (v1.26.0). Sequence alignments were constructed using DECIPHER (v2.16.1), and phylogenetic trees were built using Phangorn (v2.5.5). Visualization of the trees was performed using the online tool iTOL (v6.9.1). Exometabolite-targeted samples were analyzed with Trace Finder 4.1 software (Thermo Fisher Scientific), while untargeted metabolite data analyses were conducted using MZmine (v3.0). All visualizations, except for the trees, were created using ggplot2 (v3.5.1). Non-parametric tests were performed using the Kruskal-Wallis test, followed by two-sided Dunn's post-hoc test with Benjamini-Hochberg (BH) adjustment for multiple comparisons, implemented using the PMCMRplus (v1.9.10), dunn.test (v1.3.6), and rcompanion (v2.4.36) R packages. PERMANOVA (PERmutational Multivariate ANalysis Of VAriance using distance matrices) was performed with the adonis2() function in the vegan package (v2.6-6.1), using Bray-Curtis distances for community profiling data and Canberra distances for metabolomics data. Significance was determined at P≤0.05 and indicated by significance groups. For comparisons between two conditions, the non-parametric two-sided Wilcoxon test was applied using the wilcox.test function from the stats package (v4.2.2). Statistical significance between proportions was assessed using Fisher's exact test via the fisher.test function from the stats package (v4.2.2). The specific statistical tests used are indicated in each figure legend. Plant length measurements were performed using Fiji, and figures were assembled in Adobe Illustrator. No data were excluded from the analyses.

For manuscripts utilizing custom algorithms or software that are central to the research but not yet described in published literature, software must be made available to editors and reviewers. We strongly encourage code deposition in a community repository (e.g. GitHub). See the Nature Portfolio guidelines for submitting code & software for further information.

Data

Policy information about availability of data

All manuscripts must include a data availability statement. This statement should provide the following information, where applicable:

- Accession codes, unique identifiers, or web links for publicly available datasets
- A description of any restrictions on data availability
- For clinical datasets or third party data, please ensure that the statement adheres to our policy

Data Availability
The raw sequencing data from SynCom reconstitution experiments have been deposited in the European Nucleotide Archive (ENA) at EMBL-EBI under accession number PRJEB80329 (https://www.ebi.ac.uk/ena/browser/view/PRJEB80329). The MS raw data of the targeted and untargeted metabolomics are deposited on MassIVE at ftp://massive.ucsd.edu/v06/MSV000096804/ (doi:10.25345/C5V40KB3M). We provide a protocol in PDF format (Supplementary Protocol 1), with the latest version accessible on protocols.io (DOI : dx.doi.org/10.17504/protocols.io.36wgqd68ovk5/v2) 63. We provide the complete set of 3D printing models (Supplementary Data 1-6,18) and individual stereolithography files (Supplementary Data 7-12,19); future updates to the 3D files will be available in the online protocol in the Material section. Datasets can be accessed via GitHub (see Code Availability) or through the source data provided with this paper.

Code availability
All scripts and data sets employed in this work are available from GitHub at https://github.com/gchesneau53/Script_from_Chesneau_et_al_2025

Research involving human participants, their data, or biological material

Policy information about studies with human participants or human data. See also policy information about sex, gender (identity/presentation), and sexual orientation and race, ethnicity and racism.

Reporting on sex and gender	not applicable
Reporting on race, ethnicity, or other socially relevant groupings	not applicable
Population characteristics	not applicable
Recruitment	not applicable
Ethics oversight	not applicable

Note that full information on the approval of the study protocol must also be provided in the manuscript.

Field-specific reporting

Please select the one below that is the best fit for your research. If you are not sure, read the appropriate sections before making your selection.

☒ Life sciences ☐ Behavioural & social sciences ☐ Ecological, evolutionary & environmental sciences

For a reference copy of the document with all sections, see nature.com/documents/nr-reporting-summary-flat.pdf

Life sciences study design

All studies must disclose on these points even when the disclosure is negative.

Sample size	No statistical methods were used to pre-determine sample sizes. Statistical tests were selected based on the sample size and experimental design. Individual data points are shown for all experiments to illustrate the full range of replicates.
Data exclusions	No samples were excluded from the analyses.
Replication	All experiments included at least three replicates, each corresponding to distinct samples. For each individual MetaFlowTrain experiment, samples were run in parallel, conditions were randomly distributed across media and pumps, with each replicate using a unique microchamber or microchamber train. Peat extracts were prepared from Flowpots grown in separate boxes.
Randomization	All experiments involving plants grown on agar plates or in Flowpots were randomly distributed within the growth chambers and shuffled every week to minimize localization impact . MetaFlowTrain experiments included the random distribution of microchamber conditions across media and pumps.
Blinding	During sample harvesting and processing, we used numeric identifiers instead of full sample names for simplicity and blinding. However, sample names were necessary for statistical analysis and plotting, as group comparisons could not be accurately performed without knowing the corresponding group identities.

Reporting for specific materials, systems and methods

We require information from authors about some types of materials, experimental systems and methods used in many studies. Here, indicate whether each material, system or method listed is relevant to your study. If you are not sure if a list item applies to your research, read the appropriate section before selecting a response.

Materials & experimental systems

n/a	Involved in the study
x	☐ Antibodies
x	☐ Eukaryotic cell lines
x	☐ Palaeontology and archaeology
x	☐ Animals and other organisms
x	☐ Clinical data
x	☐ Dual use research of concern
☐	x Plants

Methods

n/a	Involved in the study
x	☐ ChIP-seq
x	☐ Flow cytometry
x	☐ MRI-based neuroimaging

Dual use research of concern

Policy information about dual use research of concern

Hazards

Could the accidental, deliberate or reckless misuse of agents or technologies generated in the work, or the application of information presented in the manuscript, pose a threat to:

No	Yes
x	☐ Public health
x	☐ National security
x	☐ Crops and/or livestock
x	☐ Ecosystems
x	☐ Any other significant area

Experiments of concern

Does the work involve any of these experiments of concern:

No	Yes
x	☐ Demonstrate how to render a vaccine ineffective
x	☐ Confer resistance to therapeutically useful antibiotics or antiviral agents
x	☐ Enhance the virulence of a pathogen or render a nonpathogen virulent
x	☐ Increase transmissibility of a pathogen
x	☐ Alter the host range of a pathogen
x	☐ Enable evasion of diagnostic/detection modalities
x	☐ Enable the weaponization of a biological agent or toxin
x	☐ Any other potentially harmful combination of experiments and agents

Plants

Seed stocks	A. thaliana ecotype Columbia-0 (Col-0)
Novel plant genotypes	None
Authentication	not applicable
